# Polymers Enhancing Bioavailability in Drug Delivery

**DOI:** 10.3390/pharmaceutics14102199

**Published:** 2022-10-16

**Authors:** Ana I. Fernandes, Angela F. Jozala

**Affiliations:** 1 CiiEM—Interdisciplinary Research Center Egas Moniz, Instituto Universitário Egas Moniz, Monte de Caparica, 2829-511 Caparica, Portugal; 2 LaMInFE—Laboratory of Industrial Microbiology and Fermentation Process, University of Sorocaba, Sorocaba 18023-000, SP, Brazil

A drug’s bioavailability, i.e., the extent to and rate at which it enters the systemic circulation, thus accessing the site of action, is largely determined by the properties of the drug. Many of the drugs currently entering the clinic are highly hydrophobic and/or present high molecular weights; others are highly sensitive and easily degrade upon administration. Pharmaceutical interventions to circumvent poor water solubility and permeability issues, as well as the physicochemical instability or degradation in the body, often rely on the use of polymers, either natural or synthetic, which confer unique properties to the dosage forms and contribute to better clinical outcomes. Therefore, polymeric excipients have been widely introduced in pharmaceutical manufacturing, for the delivery and targeting of many drugs, improving their pharmacokinetics and pharmacodynamics.

This Special Issue aims to provide an update on the state of the art and current trends in polymeric drug-delivery systems specifically designed for improving drug bioavailability. A total of 32 papers were submitted for publication, and those accepted (40.6% acceptance rate) may be found online; this Special Issue comprises 10 original research papers and 3 literature reviews from across the globe.

The majority of the papers (8) report the use of polymeric carriers (e.g., liposomes, microspheres, micelles, nanoparticles, inclusion complexes and supramolecular aggregates) for targeted or controlled delivery. Strategies such as the development and evaluation of novel polymers (some of which with stimuli-responsive attributes), solid-state modifications to enhance drugs’ solubility, the promotion of permeation across biological barriers, and polymer–protein drug conjugation are addressed.

Figure 1 summarizes the routes of administration of the systems described in this Special Issue. The parenteral and oral routes are the most prevalent (10 papers), followed by the oromucosal (buccal), topical and pulmonary routes, with 1 publication each. Unsurprisingly, drugs presenting anticancer activity or those effective against inflammation and autoimmune diseases predominate. 

A concise summary of the polymer-based drug-delivery systems and strategies presented in each paper is provided below.

Silibinin, a hepatoprotective, anticancer and chemopreventive agent with low aqueous solubility, and chemical instability, has been incorporated in PEG-modified tert-octylcalix[8]arenes, as a drug-delivery platform [1]. In the first phase, the novel PEGylated polymers were synthesized by the anionic polymerization of ethylene oxide and characterized using diffusion-ordered NMR spectroscopy. The resulting amphiphilic macromolecules consisted of a hydrophobic calixarene core and eight hydrophilic PEG chains. Inclusion complexes and supramolecular aggregates containing silibinin were then produced by a solvent-evaporation method. The nanosized self-assembled structures, which were formed above the critical micellar concentration, dramatically enhanced the solubility (>1700%) of the drug candidate due to the establishment of hydrophobic non-covalent host–guest interactions, thus promoting the drug’s solubilization. In vitro release studies, conducted by membrane dialysis under physiologically relevant conditions, showed a biphasic release of the drug: initial fast release from the aggregates, followed by a delayed drug release from the inclusion complexes, for up to 24 h. The cytotoxicity of drug-loaded and drug-free constructs against human tumor cell lines was evaluated; the constructs were biocompatible and did not compromise the antineoplastic potential of silibinin.

Simões and co-workers developed lecithin-based liposomes complexed with copolymers of Pluronic^®^ and poly(acrylic acid)/poly(N,N-dimethylaminoethyl methacrylate), for controlled drug release [2]. The copolymers, previously synthesized by atom transfer radical polymerization, stabilized the structure of the liposomes, as measured by the leakage of calcein, a fluorescent dye encapsulated in their aqueous compartment. The polymer–liposome complexes presented a homogeneous particle size in the nanometer range, low polydispersity and significantly negative surface charge, preventing agglomeration and promoting stability over time. The absence of cellular toxicity was demonstrated by the maintenance of the viability of human epithelial cells. Moreover, the polymer–liposome complexes showed pH- and temperature-responsive behavior, with higher and faster release of the marker dye. This may be of particular interest for the targeted diagnosis and treatment of diseases whose pathophysiology is characterized by changes in pH/temperature (e.g., tumors, inflammation and infection). Additionally, noteworthy is the possibility of the particles incorporating both hydrophobic and hydrophilic payloads and of presenting long plasma circulating half-lives due to the hydrophilic nature of the polymers used.

Polymer-modified liposomes, as drug-delivery systems, have been thoroughly reviewed by Cao, Dong and Chen [3]. The paper guides the reader from the early days when the first PEGylated liposomal formulation was approved by the FDA (Doxil^®^, 1995) to the most recent approaches to liposomes’ modification and marketed products. While preserving the properties of conventional liposomes (e.g., the incorporation of both hydrophobic and hydrophilic drugs, biocompatibility, tunable physicochemical and biophysical properties, the controlled release of drugs, passive targeting to tumors, and reduced drug toxicity), the surface modification of the carrier modulates its physiological properties. Polymers are mainly grafted or physically adsorbed onto the surfaces of liposomes, to increase the colloidal stability and prevent rapid uptake by the mononuclear phagocytic system and blood clearance. Specific functionalities lent by the surface polymers—e.g., poly(ethylene glycol), hyaluronic acid, chitosan and alginate—include long blood circulation times and targeting and/or stimulus-responsive features, resulting in improved drug pharmacokinetics and pharmacodynamics. The advantages and disadvantages provided by each polymer, and future research and regulatory perspectives are also addressed in this review.

S-propargyl-cysteine has recently been shown to be an outstanding endogenous hydrogen sulfide (H_2_S) donor, thus capable of alleviating the symptoms of rheumatoid arthritis, an inflammatory autoimmune disease, which is incapacitating if left untreated. However, to be clinically relevant, H_2_S must not be released instantly but rather over a period of time. The sustained release of the molecule was achieved in the work of Yu et al. [4] through the production of poly(lactic) acid microspheres. The microparticulate system was produced using a double emulsion evaporation method. The formulation that produced spherical microparticles of adequate size (≈30 μm; it induced no inflammation at the injection site) and encapsulation efficiency was chosen for further studies. The system was demonstrated to deliver H_2_S for up to 4 days in vitro and 3 days in vivo, following a single subcutaneous injection in Sprague Dawley rats. In a rat model for studying anti-inflammatory effects, the therapeutic efficacy against rheumatoid arthritis was also improved and the administration time interval was increased, compared to those of the free molecule.

Histone deacetylase inhibitors, such as MPT0B291, an azaindolysulfonamide, are a new class of antitumor agents currently under investigation; however, MPT0B291′s very low water solubility limits its clinical use and hinders its formulation for the parenteral route. To circumvent this problem, the encapsulation of MPT0B291 into human serum albumin nanoparticles was attempted [5]. Nanoparticles (≈136 nm; polydispersity index < 0.3; high encapsulation efficiency and drug loading) produced by a two-stage emulsification method remained stable for up to 4 weeks in storage, and exhibited in vitro sustained release of the drug. The cytotoxic effect on human pancreatic carcinoma cells was equivalent to that of the free drug. However, the nanoparticulate drug-delivery system provided a higher maximum tolerated intravenous single dose, with reduced side-effects on the normal cells of Balb/c mice. Additionally, in vivo pharmacokinetic studies, in the Sprague Dawley rat model, showed a 5–8-fold increase in bioavailability and a longer blood half-life (2.5 times higher than that of the free drug), which led to a significant improvement of the anticancer efficacy. The authors claim that the controlled-release, nanoscale, biocompatible, biodegradable, targeted, safe and effective injectable preparation developed may be of significant help in bringing other hydrophobic drugs to the clinic.

The effective delivery of peptide and protein therapeutics remains rather challenging and requires frequent administration by the parenteral route. The task becomes more difficult if a local, sustained, controlled delivery of the protein drug is needed. To address these problems, a novel polymer–protein conjugate with poly(ethylene glycol), capped with a high-affinity adamantane, was synthesized; the conjugate was then complexed with a cyclodextrin-based polymer by hydrophobic-driven thermodynamic interactions between the polymeric cyclodextrin cavity and the protein payload cap. Bovine serum albumin and anti-interleukin-10 monoclonal antibodies were used, respectively, as a model protein and as proof of the functionality of the system [6]. The affinity-based construct was capable of maintaining sustained drug release for up to 65 days, largely preserving both structure and protein function. The possibility of leveraging the affinity-driven loading of cyclodextrins with hydrophilic, high-molecular-mass compounds—in turn, prolonging drug release while maintaining antigen specificity (≈70%)—was demonstrated for the first time. Although in vivo experiments are warranted, significant clinical applications of the strategy, for antibody-based treatments in cancer or autoimmune diseases, are expected.

The intestinal mucosal barrier poses a challenge in oral drug delivery, and the development of dual-acting zeta-potential-amphoteric micelles has been proposed to overcome it, by allowing optimal mucopermeation and enhancing cellular uptake [7]. The rationale of the work is that, since the zeta potential of nanoemulsions is known to influence both parameters in opposite directions (cellular uptake is promoted by positively charged and permeation by negatively charged nanodroplets), a system capable of zeta-potential shifting would be beneficial. With this in mind, the study utilized mixed micelles formed by non-ionic surfactants—including Kolliphor, Labrasol (EL and RH 40) and dimethylsulfoxide as a co-solvent—as a drug-delivery method. The anchorage of excess stearic acid (SA) with a hydrophilic carboxylic acid moiety oriented in the hydrophilic shell provided micellar droplets with a high anionic density. A cationic surface was attained by using excess SA, forming hydrophobic ionic complexes with two lipophilic cationic polymers (Eudragit RS 100 and Eudragit RL 100) incorporated within the micellar hydrophobic core, and the exposure of the ammonium groups of polymers. The two types of zeta-potential-changing micellar droplets were loaded with fluorescein diacetate, a hydrophobic model drug, and the diffusion and cellular uptake through porcine intestinal mucus were investigated. The complex-loaded micellar droplets provided a significantly higher cellular uptake of the model drug than blank micelles, and showed no toxicity towards Caco-2 cells. Due to undergoing slow and time-dependent shifts in zeta potential, the modified micelles significantly enhanced the cellular uptake while preserving the mucus-permeating properties, offering dual benefits in drug delivery.

Polymeric drug-delivery systems, which have also emerged as a robust approach to enhancing oral drug bioavailability and intestinal drug absorption, are the focus of the next set of papers. In this respect, the thiolation of polymers, as a means for enhancing their mucoadhesive properties, is one of the most promising approaches to improving the therapeutic indices of drugs, by prolonging the mucosal residence time due to adhesion to mucins at the site of action; additionally, enhanced permeation across mucosa and enzymatic protection from degradation are also provided. Zaman et al. [8] describe the successful thiolation of poloxamer, an amphipathic excipient widely used in pharmacy. The thiomer obtained was evaluated regarding its physicochemical properties, biocompatibility in albino rats and adequacy as an excipient for producing compressed tablets. Tacrolimus, a poorly bioavailable BCS class II immunosuppressant drug, was used as a model. Tacrolimus-containing tablets, produced with the thiomer, showed a satisfactory drug-loading capacity, superior mucoadhesion and an improved in vitro dissolution profile (faster initial release with kinetics independent of the initial drug concentration and a diffusion-type release pattern), indicating that they were suitable for the controlled oral delivery of drugs.

A different strategy, with the same purpose of increasing drug oral bioavailability, is described in the next paper. A stable solid dispersion of the poorly water-soluble glycyrrhetinic acid (GA, a potent anti-inflammatory triterpene saponin) was produced by co-solvent evaporation. l-arginine, used as a low-molecular-weight co-former, produced co-amorphous salts of the drug, which were, in turn, added with Soluplus^®^, a matrix-forming amphiphilic polymer, to obtain the solid dispersion via hydrogen bonding or complexation reactions [9]. Above the critical micellar concentration, Soluplus^®^ produced micelles encapsulating the hydrophobic drug in the core, promoting solubility and preventing drug crystallization. The solid dispersion was characterized by different microscopic and spectroscopic methods (e.g., FTIR and XRD), and the anti-inflammatory activity was evaluated in a cellular inflammation model and in ear edema and gastric ulcer models in mice. The new oral formulation showed adequate drug loading in the polymer, with dramatically improved solubility, due to the molecular interactions established. The particle size was below 100 nm, allowing the particles to evade rapid clearance by the mononuclear phagocytic system and increasing their cytomembrane penetrability. Furthermore, the immunomodulatory effect of the drug was superior, which translated into improved anti-inflammatory activity both in vitro and in vivo. The authors conclude that this is a safe and effective method for improving the solubility and bioavailability of GA, also providing guidance for other drug candidates showing poor oral bioavailability.

The next work in the Special Issue reviews the gastrointestinal physiological challenges (e.g., poor absorption, metabolic instability, the epithelial mucus layer, intestinal motility, efflux pumps and disease) impacting the bioavailability of drugs and how chitosan-based drug-delivery systems may overcome such barriers, while changing the target sites of absorption [10]. Chitosan is a cationic, biodegradable/biocompatible, atoxic and versatile molecule that has been shown to improve the intestinal assimilation of drugs. Recent advances in the development and application of chitosan-based systems that improve intestinal drug absorption, its mechanisms, and pharmacological applications are extensively discussed. In short, chitosan (or its derivatives) nanoparticles/nanocapsules may be obtained by ionic gelation, chemical modification, and polyelectrolyte complexation methods; the oral drug absorption is improved due to protection from enzymatic degradation, mucoadhesion, efflux inhibition, antimicrobial activity, and enhanced permeation effects of the polymer, which is also capable of controlling the release of the drug. Clinical use in diabetes, cancer, infections and inflammation is envisaged as a new paradigm for nanotechnology-based treatments.

Developing new delivery systems to reduce the risk of intoxication with drugs with narrow therapeutic indices is of the utmost importance. Digoxin is one such drug that is used to treat heart failure and atrial fibrillation, and raises safety concerns, especially in the elderly. However, mucosal drug administration has recently received attention from researchers, as it avoids the hepatic first-pass effect and degradation by gastrointestinal enzymes, providing rapid drug absorption and increasing bioavailability. Additionally, polymeric nanoparticles have wide-ranging potential as carrier systems for bioactive compounds, controlling the drug release profiles and reducing degradation and toxicity. With these premises in mind, the study aimed to demonstrate the potential of sodium alginate films containing digoxin-loaded zein nanoparticles, as a buccal drug-delivery system, to reduce the number of doses and facilitate administration and a rapid onset of action [11]. Sodium alginate was selected for the film matrix, since it is a hydrophilic, biocompatible polysaccharide, with mucoadhesive properties, which may lead to an increased residence time at the site of action. The films were obtained by solvent casting, and the nanoparticles were produced by the nanoprecipitation method. Zein is not only mucoadhesive and biocompatible, but also amphiphilic and thus capable of encapsulating hydrophobic drugs, such as digoxin. In fact, digoxin was efficiently encapsulated (91%) and the particles (≈ 87 nm) were stable and monodisperse and exhibited positive charges, making them capable of interacting with the negatively charged sialic acid residues in mucin and prolonging the buccal residence time. Films were produced with and without a plasticizer (glycerol) and varying concentrations of ethanol; the system containing 10% ethanol presented a swelling profile and mechanical properties compatible with application as a buccal drug-delivery system. The medicated films also showed controlled drug release, with potential for improved therapeutic effects and compliance, with reduced side effects. Though complementary assays are necessary, the system developed seems to be a good alternative to the conventional digoxin solid dosage forms currently available on the market.

Another important strategy addressed in this Special Issue was the use of liposomal formulations to overcome resistance to antibiotics, which results in increasing difficulty in treatment. Gbian and Omri evaluated the efficacy of free and liposome-encapsulated antibiotics—gentamycin (GEN) and erythromycin (ERY)—in combination with a broad-spectrum efflux-pump inhibitor (phenylalanine-arginine β-naphthylamide—PABN)—against *Pseudomonas aeruginosa* strains [12]. Chronic and persistent infections with this opportunistic pathogen are the leading cause of death in cystic fibrosis patients. Antibiotic multiresistance may be due to the poor penetration or active removal of antibiotics from the cells by efflux pumps, justifying the use of inhibitors in this work. Liposomes were prepared by the dehydration–rehydration vesicle method and characterized with respect to size, size distribution and encapsulation efficiency; the antimicrobial activity was determined by the microbroth dilution method. The activity on *P. aeruginosa* biofilms, and the effects of sub-inhibitory concentrations on the virulence factor, quorum-sensing signals and bacterial motility were also studied. The authors showed that the liposomal encapsulation of antibiotics increased the drug penetration and therapeutic effectiveness. PABN combinations potentiated the antibiotics by reducing the minimal inhibitory and bactericidal concentrations by 4 to 32 times in total, for both GEN and ERY. In fact, liposomal antibiotics combined with PABN proved efficacious in inhibiting bacterial growth, eradicating biofilms and reducing virulence factors and motility. Further in vitro and in vivo tests are needed to fully understand the potential impact and utility of the strategy proposed for the clinical management of *P. aeruginosa* infections in cystic fibrosis.

The last paper addresses the relevance of treating chronic wounds in view of the significant burden they represent to healthcare systems and their negative impact on patients’ quality of life [13]. The wound-healing cascade and wound-care strategies, such as debridement and especially wound dressing, are reviewed in detail. The properties of the ideal wound dressing are discussed, as well as the use of nitric oxide (NO) for wound healing due to its effects, such as promoting vasodilation, cell proliferation, and angiogenesis, and antimicrobial activity. Polymers may be engineered with an array of materials, fulfilling the required properties of a wound dressing. In this respect, hydrogels, which may be used as storage and delivery matrices for NO, are extensively covered. Such NO-releasing hydrogel-based systems (presenting either physically adsorbed or chemically attached NO donors) have been proven to exhibit bactericidal properties, enhance wound healing, and promote the controlled and sustained release of NO when required. The advantages and disadvantages of NO-donor incorporation in hydrogels and the mechanisms of NO release are also presented. The need for dressings customized according to each type of wound is deemed possible to meet by adjusting the hydrogel. However, extensive characterization of the physicochemical properties, NO-release kinetics and toxicity profile upon chronic exposure will be essential in the future. 

Overall, these contributions further strengthen the role of polymers in modern drug delivery and targeting, illustrating the multiple approaches possible and unveiling what the future may bring.

## Figures and Tables

**Figure 1:**
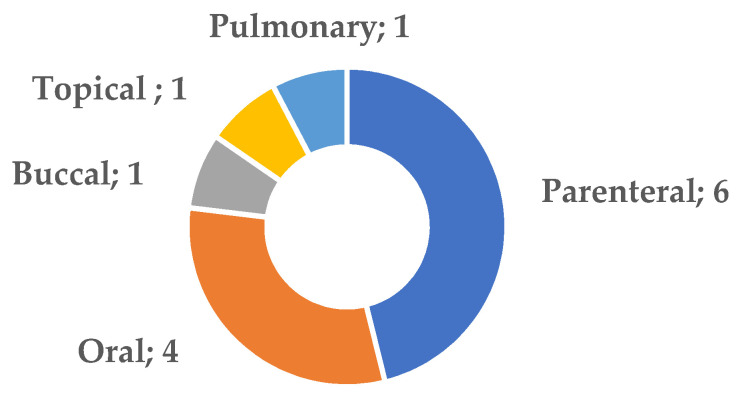
Numbers of papers published by administration route used (or intended).
